# A potentially novel overlapping gene in the genomes of Israeli acute paralysis virus and its relatives

**DOI:** 10.1186/1743-422X-6-144

**Published:** 2009-09-17

**Authors:** Niv Sabath, Nicholas Price, Dan Graur

**Affiliations:** 1 Department of Biology and Biochemistry, University of Houston, Houston, TX 77204, USA

## Abstract

The Israeli acute paralysis virus (IAPV) is a honeybee-infecting virus that was found to be associated with colony collapse disorder. The IAPV genome contains two genes encoding a structural and a nonstructural polyprotein. We applied a recently developed method for the estimation of selection in overlapping genes to detect purifying selection and, hence, functionality. We provide evolutionary evidence for the existence of a functional overlapping gene, which is translated in the +1 reading frame of the structural polyprotein gene. Conserved orthologs of this putative gene, which we provisionally call *pog *(*p*redicted *o*verlapping *g*ene), were also found in the genomes of a monophyletic clade of dicistroviruses that includes IAPV, acute bee paralysis virus, Kashmir bee virus, and *Solenopsis invicta *(red imported fire ant) virus 1.

## Background

Colony collapse disorder (CCD) is a syndrome characterized by the mass disappearance of honeybees from hives [1]. CCD imperils a global resource estimated at approximately $200 billion [2]. For example, it has been estimated that up to 35% of hives in the US may have been affected [3]. Many culprits have been suggested as causal factors of CCD, among them fungal, bacterial, and protozoan diseases, external and internal parasites, in-hive chemicals, agricultural insecticides, genetically modified crops, climatic factors, changed cultural practices, and the spread of cellular phones [1]. The Israeli acute paralysis virus (IAPV), a positive-strand RNA virus belonging to the family Dicistroviridae, was found to be strongly correlated with CCD [4]. It was first isolated in Israel [5], but was later found to have a worldwide distribution [4,6,7].

The genome of IAPV contains two long open reading frames (ORFs) separated by an intergenic region. The 5' ORF encodes a structural polyprotein; the 3' ORF encodes a non-structural polyprotein [5]. The non-structural polyprotein contains several signature sequences for helicase, protease, and RNA-dependent RNA polymerase [5]. The structural polyprotein, which is located downstream of the non-structural polyprotein, encodes two (and possibly more) capsid proteins.

Overlapping genes are easily missed by annotation programs [8], as evidenced by the fact that several overlapping genes were only detected by using the signatures of purifying selection [9-13]. Here, we apply a recently developed method for the detection of selection in overlapping reading frames [14] to the genome of IAPV and its relatives.

## Results and Discussion

In the fourteen completely sequenced dicistroviral genomes (Table 1), we identified 43 same-strand overlapping ORFs of lengths equal or greater than 60 codons on the positive strand. Ten overlapping ORFs were found in concordant genomic locations in two or more genomes. The concordant overlapping ORFs were assigned to three orthologous clusters (Table 2). The overlapping ORFs in all three clusters are phase-1 overlaps, i.e., shifted by one nucleotide relative to the reading-frames of the known polyprotein genes. Two of the orthologous clusters overlap the gene encoding the nonstructural polyprotein and one overlaps the reading frame of the structural polyprotein. (In appendix 1, we present the results concerning the overlapping ORFs on the negative strand. We note, however, that dicistroviruses are not known to be ambisense [15].)

**Table 1:** A list of completely sequenced dicistroviruses used in this study

**Name**	**Accession number**
Israel acute paralysis virus (IAPV)	GenBank:NC_009025
Acute bee paralysis virus (ABPV)	GenBank:NC_002548
Kashmir bee virus (KBV)	GenBank:NC_004807
*Solenopsis invicta *virus (SINV-1)	GenBank:NC_006559
Black queen cell virus (BQCV)	GenBank:NC_003784
Cricket paralysis virus (CrPV)	GenBank:NC_003924
*Homalodisca coagulata *virus-1 (HoCV-1)	GenBank:NC_008029
*Drosophila *C virus (DCV)	GenBank:NC_001834
Aphid lethal paralysis virus (ALPV)	GenBank:NC_004365
Himetobi P virus (HiPV)	GenBank:NC_003782
Taura syndrome virus (TSV)	GenBank:NC_003005
*Plautia stali *intestine virus (PSIV)	GenBank:NC_003779
*Triatoma *virus (TrV)	GenBank:NC_003783
*Rhopalosiphum padi *virus (RhPV)	GenBank:NC_001874

**Table 2:** Clusters of orthologous overlapping ORFs on the positive strand

**Cluster**	**Virus**	**Start of ORF**	**End of ORF**	**Length** **(nucleotides)**
A	IAPV	6589	6900	312
	ABPV	6513	6815	303
	KBV	6601	6909	309
	SINV-1	4382	4798	417

B	ABPV	5958	6227	270
	KBV	5974	6243	270

C	CrPV	2396	2614	219
	DCV	2216	2602	387
	HoCV-1	2377	2574	198
	PSIV	2333	2527	195

**Figure 1:**
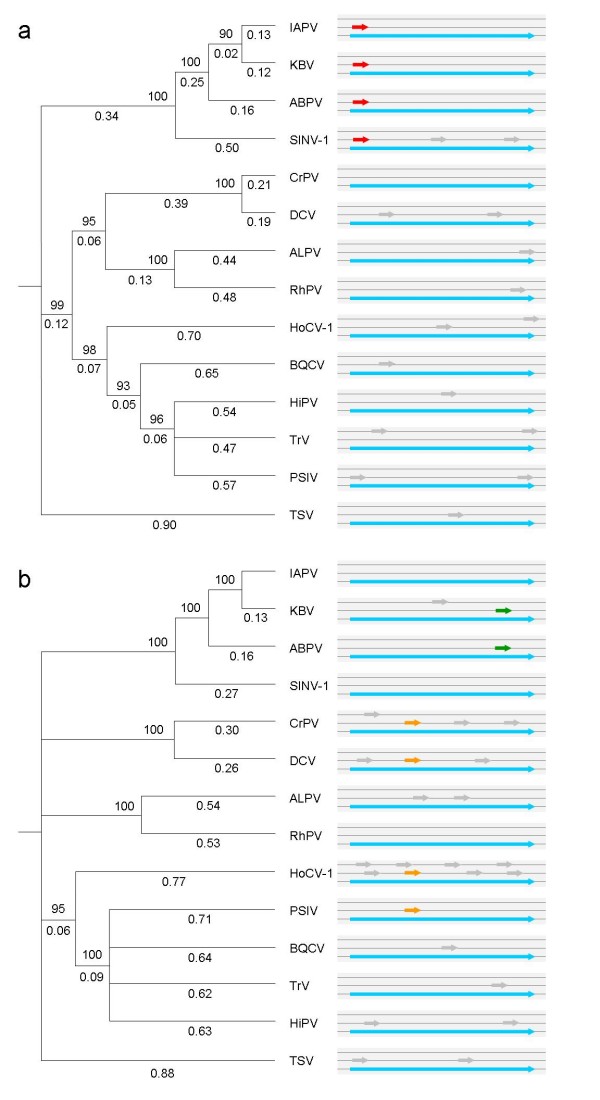
**Phylogenetic trees and schematic representation of the dicistrovirid genomes (a. structural polyprotein; b. non-structural polyprotein)**. Trees were inferred using the neighbor joining method [30] and rooted by the mid-point rooting method [31]. Numbers above and below the branches are bootstrap values (1000 replications) and branch lengths (amino-acid substitutions per site), respectively. Phylogenetic analyses were conducted with MEGA [28]. The approximate locations and sizes of the known genes (blue), overlapping hypothetical genes (red, green, and orange), and singlet ORFs (gray) are noted in the three reading frames.

We identified a strong signature of purifying selection in cluster A that contains overlapping ORFs from four genomes: IAPV, Acute bee paralysis virus (ABPV), Kashmir bee virus (KBV), and *Solenopsis invicta *virus 1 (SINV-1) [16-18]. This ORF overlaps the 5' end of the structural polyprotein gene (Figure 1A). The detection of purifying selection is based on a method for the simultaneous estimation of selection intensities in overlapping genes [14]. To ascertain that each overlapping ORF is indeed subject to selection, we used the likelihood ratio test for two hierarchical models. In model 1, we assume no selection on the overlapping ORF. In model 2, the overlapping ORF is assumed to be under selection. If model 2 fits the data significantly better than model 1 (*p *< 0.05), then the overlapping ORF is predicted to be under selection and is most probably functional. The signature of selection was identified for the ORFs in the three bee viruses (IAPV, ABPV, and KBV). The protein product of the orthologous ORF in SINV-1 could not be tested for selection because the amino acid sequence identity between the ORF from SINV-1 and the ORFs from the three bee viruses (Table 3) is lower than the range of sequence identities for which the method can be applied (65-95%).

**Table 3:** Sequence conservation in comparisons of known orthologous proteins and orthologous products of overlapping ORFs.

**Cluster**	**Genome pair**		**Identity of known proteins (%)**	**Identity of hypothetical product of overlapping ORFs (%)**
A	IAPV	ABPV	80.2	74.8
	ABPV	KBV	79.3	75.6
	IAPV	KBV	77.4	72.5
	IAPV	SINV-1	42.7	30.3
	ABPV	SINV-1	41.6	32.6
	KBV	SINV-1	36.3	29.4

B	KBV	ABPV	87.7	52.3

C	CrPV	DCV	80.3	36.1
	HoCV-1	PSIV	64.3	40.0
	DCV	HoCV-1	56.4	28.8
	CrPV	HoCV-1	48.0	31.7
	DCV	PSIV	44.2	36.4
	CrPV	PSIV	35.7	25.0

An additional indication for selection on these ORFs was obtained by comparing the degrees of conservation of the hypothetical protein sequences of the overlapping ORFs against the protein sequences of the known genes (structural and nonstructural polyproteins, Table 3). The degree of amino-acid conservation and, hence, sequence identity between orthologous protein-coding genes is influenced *ceteris paribus *by the intensity of purifying selection. If both overlapping genes are under similar strengths of selection, the amino-acid sequence identity of one pair of homologous genes would be similar to that of the overlapping pair. On the other hand, if a functional gene overlaps a non-functional ORF, the amino-acid identity between the hypothetical protein sequences of the non-functional ORFs would be much lower than that between the two homologous overlapping functional genes. We found that the degree of amino-acid conservation of the overlapping sequence identity between pairs of overlapping ORFs in cluster A is only slightly lower than that of the known gene (maximum of 12% difference between IAPV and SINV-1 in cluster A, Table 3). In contrast, the amino-acid sequence identity between ORF pairs in clusters B and C is much lower than that between the pairs of known genes (maximum of 44% difference between CrPV and DCV in cluster C, Table 3).

The signature of purifying selection on the ORFs in cluster A suggests that they may encode functional proteins. We provisionally term this gene *pog *(*p*redicted *o*verlapping *g*ene). In Figure 1, we show that *pog *is found in the genomes of four viruses that constitute a monophyletic clade, but not in any other dicistrovirid genome (Figure 1A). Its phylogenetic distribution suggests that *pog *originated before the divergence of SINV-1 from the three bee viruses. The phylogenetic distributions of the ORFs in clusters B and C (Figure 1B) are patchy. This patchiness is an additional indication that the overlapping ORFs in clusters B and C are spurious, i.e., non-functional.

**Figure 2:**
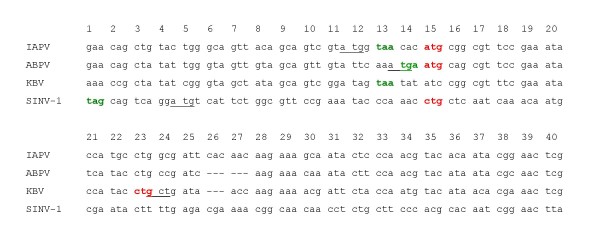
**Codon alignment of the 5' overlap region between the structural polyprotein and the hypothetical gene**. The alignment is shown in the reading frame of the hypothetical gene. The annotated initiation site of the polyproteins is underlined. The first potential initiation site (AUG or CUG) of the hypothetical genes is marked in red. The last stop codon at the +1 reading frames is marked in green.

**Figure 3:**
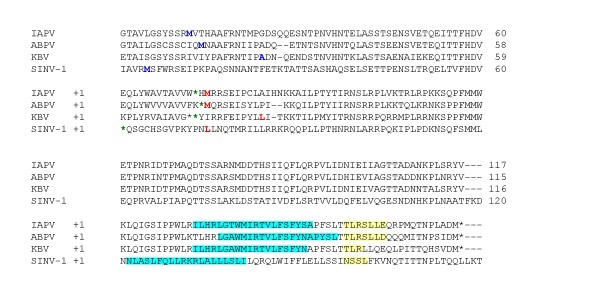
**The amino-acid alignment of the overlap region between the structural polyprotein and the hypothetical gene (+1 reading frame)**. The annotated initiation site of the polyproteins is marked in blue. The first potential initiation site (AUG or CUG) of the hypothetical genes is marked in red. The last stop codon at the +1 reading frames is marked in green. Transmembranal helixes predicted by MEMSAT [21] are marked in blue. Conserved protein kinase C phosphorylation sites predicted through My-Hits server  are marked in yellow.

An examination of the DNA alignment of *pog *(Figures 2) reveals a conservation of the first potential start codon (ATG or CTG) in the +1 reading frame in three out of the four viral genomes (IAPV, ABPV, and SINV-1). As seen in Figure 3, this conservation cannot be explained by constraints on the overlapping polyprotein, in which the corresponding site is variable and encodes different amino acids (His, Asn, and Pro, in IAPV, ABPV, and SINV-1, respectively). We note, however, that we did not find a conserved Kozak consensus sequence [19] upstream of the potential initiation site. This situation is similar to that described in [13].

A protein motif search resulted in several matches, all with a weak score. Two patterns were found in all four proteins: (1) a signature of rhodopsin-like GPCRs (G protein-coupled receptors), and (2) a protein kinase C phosphorylation site (Figure 3). Prediction of the secondary structures [20] suggests that the proteins contain two conserved helix domains, separated by 3-5 residues (except for SINV-1, in which one long domain is predicted), at the C-terminus (Figure 3). A search for transmembrane topology [21] indicates that the longer helix may be a transmembranal segment (Figure 3). Although viruses often use GPCRs to exploit the host immune system through molecular mimicry [22-25], the lengths of the proteins encoded by *pog *are shorter than the average virus-encoded GPCR. Therefore, these proteins may have a different function.

## Conclusion

In this note, we provide evolutionary evidence (purifying selection) for the existence of a functional overlapping gene, *pog*, in the genomes of IAPV, ABPV, KBV, and SINV-1. To our knowledge, this putative gene, whose coding region overlaps the structural polyprotein, has not been described in the literature before.

## Methods

### Sequence Data, Processing, and Analysis

Fourteen completely sequenced dicistrovirid genomes were obtained from NCBI (Table 1). Each genome was scanned for the presence of overlapping ORFs. We used BLASTP [26] with the protein sequences of the known genes to identify matches of orthologous overlapping ORFs (E value < 10^-6^). Matching overlapping ORFs were assigned into clusters. Within each cluster, we aligned the amino-acid orthologs by using the sequences of the known genes as references. If alignment length of the overlapping sequence exceeded 60 amino-acids, and if the amino-acid sequence identity among the hypothetical genes within a cluster was higher than 65%, we tested for selection on the hypothetical gene (see below).

We aligned the protein sequences of the two polyproteins with CLUSTAW [27] as implemented in the MEGA package [28]. Alignment quality was confirmed using HoT [29]. We reconstructed two phylogenetic trees (one for each polyprotein) by applying the neighbor joining method [30], as implemented in the MEGA package [28]. Trees were rooted by the mid-point rooting method [31] and confidence of each branch was estimated by bootstrap with 1000 replications.

### Detection of Selection in Overlapping Genes

We used the method of Sabath et al. [14] for the simultaneous estimation of selection intensities in overlapping genes. This method uses a maximum-likelihood framework to fit a Markov model of codon substitution to data from two aligned homologous overlapping sequences. To predict functionality of an ORF that overlaps a known gene, we modified an existing approach for predicting functionality in non-overlapping genes [32]. Given two aligned orthologous overlapping sequences, we estimate the likelihood of two hierarchical models. In model 1, there is no selection on the ORF. In model 2, the ORF is assumed to be under selection. The likelihood-ratio test is used to test whether model 2 fits the data significantly better than model 1, in which case, the ORF is predicted to be under selection and most probably functional.

### Motifs

We looked for motifs within the inferred protein sequences encoded by the overlapping ORF by using the motif search server  and the My-Hits server  with the following motif databases: PRINTS [33], PROSITE [34], and Pfam [35]. We used PSIPRED [20] to predict secondary structure, and MEMSAT [21] to predict transmembrane protein topology.

## Competing interests

The authors declare that they have no competing interests.

## Authors' contributions

NS carried out the analysis and wrote the draft manuscript. NP performed the motif search. DG and NP contributed to the interpretation of the results and the final version.

All authors have read and approved the manuscript.

## Appendix 1

### Overlapping ORFs on the negative strand

In the fourteen completely sequenced dicistroviruse genomes (Table 1), we identified 240 overlapping ORFs of length equal or greater than 60 codons on the negative strand. Of the 240 ORFs, 113 were found in concordant genomic locations in two or more genomes. The concordant overlapping ORFs were assigned into 29 clusters (Additional file 1). There are 9, 1, and 19 clusters in phase 0, 1, and 2, respectively. The cluster size ranges from 2 to 9. In two clusters, 5 and 10, both in phase 2, there is a weak signature of selection. However, this signature seems to be a false positive, which was driven by the unique structure of opposite-strand phase-2 overlap (Additional file 2). In this structure, codon positions one and two of one gene match codon positions two and one of the overlapping gene. This structure leads to a situation where most changes are either synonymous or nonsynonymous in both overlapping genes and occasionally, to false signal of purifying selection on the overlapping ORF. In addition, one of the clusters (cluster 10) does not constitute a monophyletic clade, and is, therefore, unlikely to be functional. We therefore conclude that dicistroviruses most probably do not encode proteins on the negative strand.

## Supplementary Material

Additional file 1 **Clusters of orthologous overlapping ORFs on the negative strands of dicistrovirid genomes.** Click here for file

Additional file 2 **The corresponding codon positions of overlapping genes in opposite-strand phase-2.** First and second codon positions, in which ~5% and 0% of the changes are synonymous, are marked in red. Third codon positions, in which ~70% of the changes are synonymous, are marked in blue. Click here for file
