# Supplementary material for: A potentially novel overlapping gene in the genomes of Israeli acute paralysis virus and its relatives
Source: Virol J. 2009 Sep 17;6:144. doi: 10.1186/1743-422X-6-144 (PMC2754452; doi:10.1186/1743-422X-6-144)
Supplement: Additional file 1 — Clusters of orthologous overlapping ORFs on the negative strands of dicistrovirid genomes. [file 1743-422X-6-144-S1.DOC]

**Additional file 1:** Clusters of orthologous overlapping ORFs on the negative strands of dicistrovirid genomes.

| Cluster | Virus | Phase | Start | End | Length |
| --- | --- | --- | --- | --- | --- |
| 1 | IAPV | 1 | 913 | 1131 | 219 |
| 1 | ABPV | 1 | 975 | 1175 | 201 |
| 1 | KBV | 1 | 1006 | 1191 | 186 |
| 2 | IAPV | 2 | 902 | 1087 | 186 |
| 2 | ABPV | 2 | 1042 | 1593 | 552 |
| 2 | IAPV | 2 | 1334 | 1630 | 297 |
| 2 | KBV | 2 | 1454 | 1690 | 237 |
| 3 | IAPV | 2 | 1634 | 1906 | 273 |
| 3 | KBV | 2 | 1724 | 1927 | 204 |
| 3 | ABPV | 2 | 1747 | 2031 | 285 |
| 4 | IAPV | 2 | 2507 | 2755 | 249 |
| 4 | ABPV | 2 | 2509 | 2697 | 189 |
| 4 | SINV-1 | 2 | 276 | 581 | 306 |
| 4 | HiPV | 2 | 2940 | 3125 | 186 |
| 4 | RhPV | 2 | 2445 | 2795 | 351 |
| 4 | TrV | 2 | 2723 | 2962 | 240 |
| 5 | IAPV | 2 | 4070 | 4510 | 441 |
| 5 | KBV | 2 | 4082 | 4375 | 294 |
| 5 | ABPV | 2 | 3982 | 4275 | 294 |
| 5 | SINV-1 | 2 | 1902 | 2186 | 285 |
| 6 | IAPV | 2 | 4694 | 4948 | 255 |
| 6 | KBV | 2 | 4706 | 4990 | 285 |
| 7 | IAPV | 2 | 5696 | 5941 | 246 |
| 7 | SINV-1 | 2 | 3570 | 3761 | 192 |
| 7 | CrPV | 2 | 5376 | 5558 | 183 |
| 8 | IAPV | 0 | 1224 | 1421 | 198 |
| 8 | ABPV | 0 | 1127 | 1318 | 192 |
| 8 | KBV | 0 | 1107 | 1349 | 243 |
| 9 | IAPV | 0 | 5859 | 6086 | 228 |
| 9 | RhPV | 0 | 5983 | 6228 | 246 |
| 9 | ALPV | 0 | 6030 | 6305 | 276 |
| 9 | ABPV | 0 | 5957 | 6307 | 351 |
| 9 | IAPV | 0 | 6111 | 6395 | 285 |
| 9 | KBV | 0 | 6102 | 6305 | 204 |
| 9 | SINV-1 | 0 | 3811 | 3996 | 186 |
| 9 | HoCV-1 | 0 | 5325 | 5537 | 213 |
| 10 | IAPV | 2 | 7601 | 7792 | 192 |
| 10 | KBV | 2 | 7436 | 7783 | 348 |
| 10 | ABPV | 2 | 7426 | 7761 | 336 |
| 10 | SINV-1 | 2 | 5805 | 6104 | 300 |
| 10 | TSV | 2 | 7729 | 7998 | 270 |
| 11 | IAPV | 2 | 8471 | 8677 | 207 |
| 11 | ABPV | 2 | 8383 | 8571 | 189 |
| 12 | IAPV | 0 | 7614 | 8099 | 486 |
| 12 | KBV | 0 | 7890 | 8102 | 213 |
| 12 | SINV-1 | 0 | 5776 | 6141 | 366 |
| 13 | IAPV | 0 | 8103 | 8402 | 300 |
| 13 | BQCV | 0 | 7094 | 7405 | 312 |
| 13 | TrV | 0 | 7366 | 7575 | 210 |
| 14 | IAPV | 0 | 8406 | 8600 | 195 |
| 14 | KBV | 0 | 8352 | 8594 | 243 |
| 15 | KBV | 2 | 3239 | 3571 | 333 |
| 15 | ABPV | 2 | 3289 | 3477 | 189 |
| 16 | KBV | 2 | 5168 | 5434 | 267 |
| 16 | ABPV | 2 | 5068 | 5250 | 183 |
| 16 | SINV-1 | 2 | 3090 | 3485 | 396 |
| 16 | PSIV | 2 | 4716 | 5006 | 291 |
| 16 | TSV | 2 | 5537 | 5764 | 228 |
| 16 | TrV | 2 | 4646 | 4864 | 219 |
| 16 | HiPV | 2 | 5130 | 5327 | 198 |
| 17 | KBV | 2 | 6821 | 7090 | 270 |
| 17 | ABPV | 2 | 6727 | 6996 | 270 |
| 18 | KBV | 2 | 8609 | 8896 | 288 |
| 18 | ABPV | 2 | 8575 | 8853 | 279 |
| 18 | SINV-1 | 2 | 6897 | 7376 | 480 |
| 19 | ABPV | 2 | 2035 | 2340 | 306 |
| 19 | SINV-1 | 2 | 28 | 269 | 242 |
| 19 | DCV | 2 | 2043 | 2231 | 189 |
| 19 | RhPV | 2 | 2073 | 2441 | 369 |
| 19 | CrPV | 2 | 2004 | 2258 | 255 |
| 19 | ALPV | 2 | 1571 | 2278 | 708 |
| 19 | BQCV | 2 | 1926 | 2123 | 198 |
| 19 | RhPV | 2 | 1617 | 2051 | 435 |
| 20 | ABPV | 2 | 5872 | 6141 | 270 |
| 20 | ALPV | 2 | 6218 | 6424 | 207 |
| 20 | CrPV | 2 | 5562 | 5786 | 225 |
| 20 | TSV | 2 | 6254 | 6436 | 183 |
| 20 | PSIV | 2 | 5643 | 5825 | 183 |
| 21 | ABPV | 0 | 5303 | 5539 | 237 |
| 21 | BQCV | 0 | 4609 | 4923 | 315 |
| 21 | SINV-1 | 0 | 3034 | 3282 | 249 |
| 21 | TrV | 0 | 4938 | 5150 | 213 |
| 21 | HoCV-1 | 0 | 4596 | 4877 | 282 |
| 21 | ALPV | 0 | 5286 | 5582 | 297 |
| 21 | RhPV | 0 | 5392 | 5583 | 192 |
| 21 | TrV | 0 | 4575 | 4781 | 207 |
| 21 | PSIV | 0 | 5086 | 5280 | 195 |
| 22 | SINV-1 | 0 | 169 | 387 | 219 |
| 22 | DCV | 0 | 2152 | 2397 | 246 |
| 22 | CrPV | 0 | 2293 | 2505 | 213 |
| 22 | RhPV | 0 | 2332 | 2586 | 255 |
| 22 | KBV | 0 | 2550 | 2756 | 207 |
| 22 | HiPV | 0 | 2806 | 3057 | 252 |
| 22 | ALPV | 0 | 2142 | 2456 | 315 |
| 23 | CrPV | 0 | 4063 | 4293 | 231 |
| 23 | RhPV | 0 | 4612 | 4836 | 225 |
| 23 | ALPV | 0 | 4692 | 4877 | 186 |
| 24 | CrPV | 2 | 6402 | 6641 | 240 |
| 24 | BQCV | 2 | 5995 | 6186 | 192 |
| 24 | PSIV | 2 | 6291 | 6578 | 288 |
| 25 | DCV | 2 | 7331 | 7531 | 201 |
| 25 | ALPV | 2 | 7806 | 8033 | 228 |
| 26 | DCV | 2 | 7718 | 7957 | 240 |
| 26 | RhPV | 2 | 8456 | 8752 | 297 |
| 27 | DCV | 0 | 6405 | 6611 | 207 |
| 27 | BQCV | 0 | 5834 | 6226 | 393 |
| 27 | HoCV-1 | 0 | 6176 | 6388 | 213 |
| 28 | PSIV | 2 | 7674 | 7910 | 237 |
| 28 | BQCV | 2 | 7366 | 7548 | 183 |
| 28 | TrV | 2 | 7701 | 7940 | 240 |
| 28 | HiPV | 2 | 8179 | 8364 | 186 |
| 29 | BQCV | 2 | 4107 | 4376 | 270 |
| 29 | HiPV | 2 | 4746 | 4988 | 243 |
