# Supplementary figures and images for: A potentially novel overlapping gene in the genomes of Israeli acute paralysis virus and its relatives
Source: Virol J. 2009 Sep 17;6:144. doi: 10.1186/1743-422X-6-144 (PMC2754452; doi:10.1186/1743-422X-6-144)

## Slide 1
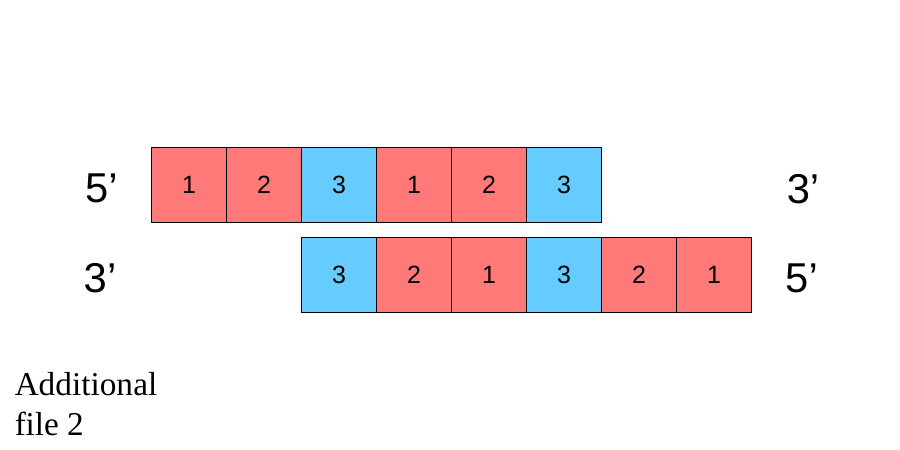

| 1 | 2 | 3 |
| --- | --- | --- |
| 1 | 2 | 3 |
| --- | --- | --- |
5’
3’
| 3 | 2 | 1 |
| --- | --- | --- |
| 3 | 2 | 1 |
| --- | --- | --- |
3’
5’
Additional file 2

Supplement: Additional file 2 — The corresponding codon positions of overlapping genes in opposite-strand phase-2. First and second codon positions, in which ~5% and 0% of the changes are synonymous, are marked in red. Third codon positions, in which ~70% of the changes are synonymous, are marked in blue. [file 1743-422X-6-144-S2.PPT]
